# Un-personalized medicine: the challenges of vaccines as a single medicine for a whole population

**DOI:** 10.1093/cei/uxaf062

**Published:** 2025-09-17

**Authors:** John S Tregoning, Vanessa Sancho-Shimizu

**Affiliations:** Department of Infectious Disease, Imperial College London, London, UK; Department of Infectious Disease, Imperial College London, London, UK

**Figure 1.**
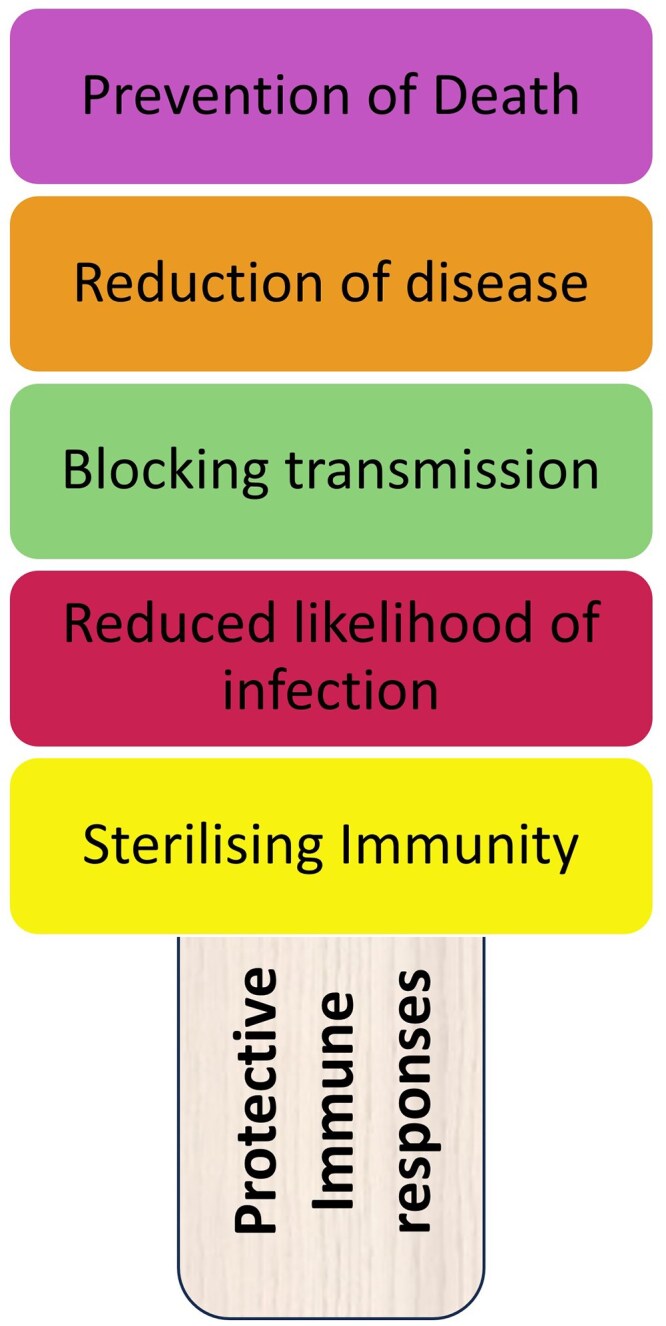
Layers of protection offered by vaccination. There is a spectrum of protection that vaccines can provide. The ideal is for sterilizing immunity, by which we mean the prevention of infection ever occurring. More broadly, a vaccine may reduce the likelihood of infection occurring, reducing the attack rate or increasing the dose of pathogen required to breach the immune system. In parallel, vaccines may reduce the amount of pathogen carried and therefore transmitted to other people. More broadly, vaccines can reduce disease and death without necessarily reducing infection or pathogen burden. All of these are beneficial

The simple message is that vaccines have had an enormous beneficial impact of in reducing infection, disease and death. But vaccines themselves are not simple, the last 5 years have (re)taught us how incredibly complex they are. A critical consideration is that vaccines are both an individual drug and a population level intervention. At the individual level, consumers want a risk-free product with minimal side effects. The ideal vaccine would provide lifelong, sterilising immunity, preventing infection from ever taking hold in the first place, or if infection occurs rapidly clearing the pathogen with no symptoms. However, from a public health perspective, different outcomes, and endpoints might be more of a priority. At the public health level, the population outcome is a priority. Reducing severe disease—which puts a burden on hospital beds, especially intensive care units and preventing death may be prioritized over reducing infection. However, if infectious spread can be reduced, so much the better as this increases value for money (Fig. 1). If one jab saves two lives, what is not to like. Ultimately, population level effects also benefit the individual—by reducing healthcare burden, freeing up resources that can be deployed elsewhere.

Vaccines sit in the realm of preventative medicine and as such need to be administered to a large number of people. And people are heterogeneous which affects all aspects of vaccination and the responses to it. There is no drug or substance to which human responses are uniform; for example a recent report showed that 21 children had acute adverse events following the consumption of something as innocuous as slushed ice drinks [1]. So, with a complex biological intervention like vaccines, there will be some people in whom there is no effect at all and others who will have severe adverse effects. One of the striking things is that adverse effects do not necessarily correlate with immunogenicity. Getting a vaccine to sit in the sweet spot of the greatest good for the greatest number is no mean feat, especially as responses are shaped by infection and immunization history.

A major challenge is that very rare events will only become apparent when larger numbers of people are inoculated. If this is done at speed and scale, such as during a pandemic, then rare adverse events may cluster together—for example during the rollout of the COVID-19 vaccine, there were 165 844 doses (Our World In Data) administered on the first day of the program (11 January 2021), which is four times as many as the whole Pfizer/BioNTech Phase III trial in a single day. At the peak, 49.6 million doses were being administered worldwide in a single day. Tools to monitor adverse events and how to manage them are an important part of wide scale vaccine rollout.

We commissioned this series of review articles to explore some of the aspects of vaccines as population medicines. The authors represent views from a diverse range of settings: academia, public health agencies, clinical immunology, funders, and biotech start-ups. The reviews explore topics that contribute to variations in efficacy across a population.

One of the biggest drivers of efficacy is host genetics. The article by Karp-Tatham *et al.* [2] evaluates how human genetic variation shapes vaccine responses, influencing both immunogenicity and the risk of rare adverse events. Studies, including those during the COVID-19 pandemic, revealed links between specific HLA alleles and SARS-CoV-2 vaccine responses, offering insight into the potential for personalized vaccines. However, challenges persist, including underpowered studies, reliance on limited phenotypes, and difficulty in accessing accurate immunization records. Research must evolve toward larger, more diverse cohorts, deeper immune profiling, and integration of genetic and functional data.

Host genetics also influences reactogenicity. Peters *et al.* [3] show that vaccination has long prevented disease and death, but individuals with inborn errors of immunity (IEI) or secondary immunodeficiencies often have reduced vaccine responses. The COVID-19 pandemic offered key insights into vaccine performance in these groups, showing partial protection is possible. Tailored strategies—such as extra doses, heterologous schedules, or timing around immunosuppressive treatments—can enhance efficacy. In cases of poor vaccine response, passive immunization, monoclonal antibodies, or immunoglobulin therapy offer alternatives. Lessons from IEI patients inform broader immunization strategies, ensuring that vulnerable populations benefit from vaccination efforts and helping to shape inclusive, individualized immunization programs.

An important corollary of this is that one method to prevent infection of individuals with IEI is to ensure high levels of herd immunity against the circulating pathogens. When some individuals make poor responses or are possibly at greater risk of adverse effects, there is a moral case to provide a blanket of protection by immunizing those that can. Vaccines can be an altruistic intervention. This can be immediately altruistic, such as vaccination in pregnancy for RSV or pertussis where the benefit is much more for the unborn child than the mother. But it can also be societally altruistic—if herd immunity for a virus such as measles is high, then individuals who cannot receive vaccines are still protected.

Each of us is much more than just our genes. Environment, previous and ongoing pathogen exposure, nutrition level all influence vaccine responses. This is particularly marked in low and middle income countries and the responses to oral vaccination. Gagandeep Kang *et al.* [4] describe geographic disparities impacting oral vaccine performance. They focus on the three licensed oral vaccines rotavirus, poliovirus and cholera, comparing differences in effectiveness in different countries—for example rotavirus vaccines have >85% effectiveness against hospitalization in infants in countries with low child mortality, but only ∼65% effectiveness in countries with high child mortality. They explore maternal, host, and environmental factors that contribute to this disparity. There is no single golden bullet to improve effectiveness in low income countries. The authors caution that different factors may have different levels of influence for different pathogens and different populations. This links back to the review by Bolze and Knight who also stressed the need for expanding research in low-resource settings to improve global relevance and understanding of vaccine efficacy across populations.

In order to better understand population level effects, we need two things large cohorts and statistical tools. Kirsebom *et al.* [5] describe England’s first vaccine register, that was established during the COVID-19 pandemic. They show how it enables timely estimates of real-world vaccine effectiveness and adverse effects; which in turn could contribute to programmatic evaluation in near real-time leading to rapid policy decisions.

One aspect that emerges is that maybe vaccines should not be a one size fits all and that more tailored approaches could be more appropriate—precision vaccinology. To move from population vaccinology different statistical tools are required. Janani *et al.* [6] give a primer on the new (and not so new) statistical approaches used in vaccine trial design that can accelerate their deployment.

There are obviously challenges to precision vaccinology, not least cost. But it has been made to work for influenza virus, where there is one vaccine for children (live attenuated influenza vaccine), one for healthy adults (low dose) and two vaccines available for the elderly (high dose or adjuvanted). An enabling factor in this is a correlate of protection that can be used as a surrogate measure. One aspect that has come under scrutiny is the question of what makes an appropriate control for vaccine studies. As with other drugs, the control will depend upon the pre-existing landscape and the question being asked. For new vaccines targeting a pathogen against which there is no vaccine, then a placebo control is appropriate; this may be an inert control or something with an equivalent degree of reactogenicity to ensure that participants do not alter their risk behaviour based on whether they believe they are vaccinated. However, if there is a licensed vaccine with known efficacy, this must be used as the correct control, because to give a placebo in these instances would not provide any protection to the trial participants and therefore knowingly put them at increased risk of infection.

We write this at a very strange time for vaccines. We now have the tools to prevent nearly every infectious disease, but there is a perception of increased reluctance in their uptake. At the time of writing there was a measles outbreak in the USA, with 1001 confirmed measles cases and 3 deaths spread across 31 states (May 2025), a pernicious infection that is entirely vaccine preventable. Whether the noisy contingent of true anti-vaxers is representative of a wider reluctance to vaccinate is unclear. A recent study from the UK indicated that the majority of mothers who did not get the RSV vaccine was because of logistical or access issues rather than ideological [7]. Ultimately, there has always been some resistance to vaccination, dating back to the days of Jenner, which puts a premium on clear communication of the benefits (and risks) of vaccination.

Overall, we hope this series of review articles helps contextualize vaccines as population medicines. Moving forwards, more precise approaches could be developed, based on different manufacturing scale, more focused clinical trial design and a better landscape of host responses based on genetics and environmental exposure.
